# Spatiotemporal response of photosynthetic characteristics in Epimedium pubescens to understory environmental factors across three agroforestry systems

**DOI:** 10.3389/fpls.2026.1796904

**Published:** 2026-05-05

**Authors:** Doudou Li, Hongbiao Zhang, Dingmei Wen, Fengmei Suo, Baolin Guo

**Affiliations:** Key Laboratory of Bioactive Substances and Resources Utilization of Chinese Herbal Medicines, Ministry of Education, Institute of Medicinal Plant Development, Chinese Academy of Medical Sciences & Peking Union Medical College, Beijing, China

**Keywords:** environmental factors, Epimedium pubescens, photosynthetic characteristics, structural equation modeling, understory cultivation

## Abstract

**Introduction:**

The sustainable utilization of traditional Chinese medicine resources highlights the dual ecological and economic value of cultivating *Epimedium pubescens* in forest understories. Nevertheless, the spatiotemporal dynamics of its photosynthetic physiology and the primary regulatory factors under varying tree canopy conditions remain insufficiently characterized.

**Methods:**

This study examined the physiological responses of *E. pubescens* story by monitoring its photosynthetic traits and key environmental factors across three artificial forest types (*Phellodendron amurense, Phoebe zhennan, and Camptotheca acuminata*) during the growing season (April–December), with plants assessed at different planting positions (inter-row vs. under-canopy). analyze.

**Results:**

The results showed that spatiotemporal differences in the understory microenvironment were regulated by canopy characteristics and stand density, with solar radiation being the key factor driving spatial variation. Diurnal dynamics of net photosynthetic rate (*P*_n_) and stomatal conductance (*g*_s_) showed significant spatial differences across the three forest stands. At the monthly scale, photosynthetic characteristics varied by tree species: in *P. zhennan* forests, *P*_n_ of inter-row plants was significantly higher than that of under-canopy plants throughout the growing season; in *P. amurense* and *C. acuminata* forests, significant positional differences in *g*_s_ occurred in autumn and summer. Variance decomposition and structural equation modeling further revealed that *P*_n_ was primarily driven by solar radiation at both diurnal (R² = 0.524) and seasonal scales (independent explanatory rate 34.3%), while *g*_s_ was mainly regulated by vapor pressure deficit at the diurnal scale and by air humidity at the seasonal scale (β = 0.49.

**Discussion:**

This study provides a physiological and ecological basis for canopy light transmittance regulation and precision cultivation of *E. pubescens* and similar crops under artificial forests.

## 1 Introduction

*Epimedium pubescens* holds a prominent place in traditional Chinese medicine, valued for its properties in tonifying kidney Yang and strengthening tendons and bones. The continued high demand for this plant in clinical and health applications has spurred its widespread cultivation (Shen et al., 2022; Liu et al., 2024; An et al., 2025; Zheng et al., 2025). Yet, expanding its production presents two main obstacles: first, the scarcity of arable land amid policies that safeguard staple food security; and second, the well-documented challenges associated with long-term monocropping—such as soil degradation, increased disease pressure, and declining medicinal quality (Zeeshan Ul Haq et al., 2023; Liu et al., 2023)—have highlighted the urgent need for more sustainable cultivation models, such as agroforestry. These pressing issues highlight the need for cultivation approaches that are both resource−efficient and ecologically sound.

One promising avenue is the adoption of agroforestry, where trees and medicinal plants are combined in space and time. These integrated forest−herb systems can boost land productivity and economic returns per unit area while also delivering ecological gains—such as moderated microclimates, enhanced biodiversity, and greater overall system stability (Brown et al., 2018; Maia et al., 2021; Li et al., 2025). As a result, they are increasingly seen as a key strategy for securing a sustainable supply of medicinal−plant resources. Earlier work by our group has shown that the yield and active−compound content of *E. pubescens* can vary considerably depending on the intercropping system used (Zhang et al., 2025b, 2026). What remains unclear, however, are the specific environmental and physiological mechanisms that give rise to these differences.

In such systems, the structure of the overstory canopy is the principal driver of understory conditions. Light—the essential energy source for photosynthesis, biomass build−up, and secondary metabolism—shows particularly marked variation in both space and time. Its intensity, quality, and duration fundamentally shape the survival, growth, and adaptive responses of understory plants (Jia et al., 2021; Su et al., 2024). Because tree species differ in architecture, they intercept, reflect, and transmit sunlight in distinct ways, creating complex, dynamic light environments below the canopy (Matsuo et al., 2021). This heterogeneity in light supply directly steers the photosynthetic adjustment of understory species, prompting changes in stomatal behavior, photosynthetic−pigment profiles, enzyme activity, and carbon−assimilation pathways—all of which eventually influence how biomass is partitioned and how medicinally active compounds accumulate (Yue et al., 2021; Singh et al., 2025).

*E. pubescens* itself is a classic shade−tolerant plant, naturally found at forest edges or beneath open canopies, which points to a specific adaptation to understory light regimes (Yang et al., 2022). Pot−based experiments under controlled conditions suggest that, over a moderate light range (about 55–73 μmol·m^-2^·s^-1^), higher irradiance promotes plant growth, photosynthetic rates, and flavonoid accumulation. Beyond this range, however, photosynthetic efficiency declines, accompanied by reduced flavonoid content (Pan et al., 2021; Ma et al., 2023; Zhang et al., 2025a). While valuable, such studies typically use fixed light levels that cannot replicate the continuous gradients and pronounced diurnal or seasonal dynamics found in real forest understories. They also rarely consider how light interacts with other co−varying factors—such as air temperature, humidity, and vapor−pressure deficit (VPD). For *E. pubescens*, several key questions therefore remain open: How does its photosynthetic physiology adjust to the spatially and temporally variable light resources generated by different stand structures? How does photosynthetic efficiency compare between planting positions—for example, between tree rows versus directly under the crown? And through which pathways, direct or indirect, do microenvironmental factors regulate its photosynthetic output and biomass accumulation? Answering these questions is essential for designing and managing *E. pubescens* intercropping systems with greater precision.

To address this gap, we carried out a field study in three common plantation−forest types in southern China, each with distinctive canopy features: *Phellodendron amurense*, *Phoebe zhennan*, and *Camptotheca acuminata*. In each stand, *E. pubescens* was planted in two representative positions: between tree rows and directly under the tree crown. The novelty of this study lies in its dual-timescale approach: we simultaneously captured instantaneous photosynthetic responses through high-resolution diurnal measurements, and long-term acclimation patterns through seasonal monitoring. This integrated temporal framework allows us, for the first time, to disentangle how the same environmental factors regulate photosynthesis differently across time scales—a critical but previously overlooked aspect in medicinal plant agroforestry research. Our work had three specific aims: (1) to quantify the diurnal and seasonal dynamics of key microclimatic variables—total solar radiation (R), air temperature, relative humidity, and VPD—with particular attention to heterogeneity in the light environment; (2) to simultaneously measure key leaf−level photosynthetic parameters (net photosynthetic rate, *P*_n_, and stomatal conductance, *g*_s_) in order to clarify how *E. pubescens* responds to and acclimates under variable light conditions; and (3) to use a combination of correlation analysis, regression modelling, and structural equation modelling (SEM) to disentangle the independent and interactive contributions of light and other microclimatic factors to photosynthesis and aboveground biomass accumulation in *E. pubescens*, thereby revealing the internal physio−ecological regulatory network. Through this approach, we elucidate how stand structure—by shaping light−resource heterogeneity—affects the photosynthetic production of an understory medicinal plant. The results offer a scientific basis for optimizing tree−species selection, spatial configuration, and management practices in *E. pubescens* intercropping systems, with broader implications for advancing the sustainable cultivation of medicinal plants.

## 2 Materials and methods

### 2.1 Description of the experimental site

The study was carried out in a cultivated field of *Epimedium pubescens* located in Taiping Town (Shawan District, Leshan City, Sichuan Province, China; 103°35′ E, 29°20′ N). The site experiences a subtropical monsoon climate, characterized by mean annual temperature of 16.5 °C, annual precipitation of approximately 1800 mm, sunshine duration around 1000 hours per year, and a frost−free period of about 280 days. The soil is classified as a loam (silty loam) with pH 6.5. Key soil chemical properties were as follows: organic matter content, 18.0 g·kg^-1^; alkali−hydrolyzable nitrogen, 0.9 g·kg^-1^; and available phosphorus, 7.6 mg·kg^-1^. Prior to the establishment of *E. pubescens* understory planting, the experimental sites were simple plantation forests with no understory crops. To ensure adequate soil fertility for the *E. pubescens* plantation, fully decomposed cattle manure was applied as a basal fertilizer at a rate of 22.5 t·ha^-1^ during land preparation in October 2022. Throughout the experimental period, all plots were managed under rainfed conditions without supplemental irrigation. Uniform weed control measures were implemented across all stands and planting positions, as needed, to minimize competition from herbaceous vegetation.

### 2.2 Experimental design

The plant material used in this study was the one-year-old ‘Guìtóng róumáo 1 hào’ (GT-1), a superior clonal line of *E. pubescens* previously selected by our research team (Yang et al., 2024). This line is characterized as “high-yielding” based on its significantly greater aboveground biomass accumulation and superior sprouting capacity compared to local wild populations in preliminary trials. It is designated as “high-quality” because its total flavonol glycoside content (epimedin A, B, C, and icariin) consistently exceeds the Chinese Pharmacopoeia standard by 4–6 times, as determined by HPLC analysis. Samples were collected from the *Epimedium* planting base in Leshan City, Sichuan Province. Seedlings were healthy and pest-free, with a height of 20–25 cm and 6–7 leaves per plant. Initial planting density was 16 plants·m^-^². One month after transplantation (December 2022), survival rate exceeded 90% across all forest types and planting positions, with no significant mortality thereafter. In November 2022, the clone was established within three distinct types of plantation forests: *Phellodendron amurense* (HB), *Phoebe zhennan* (NM), and *Camptotheca acuminata* (XS). The total planting areas were approximately 2000 m² (HB), 1333 m² (NM), and 667 m² (XS).

**Figure 1:**
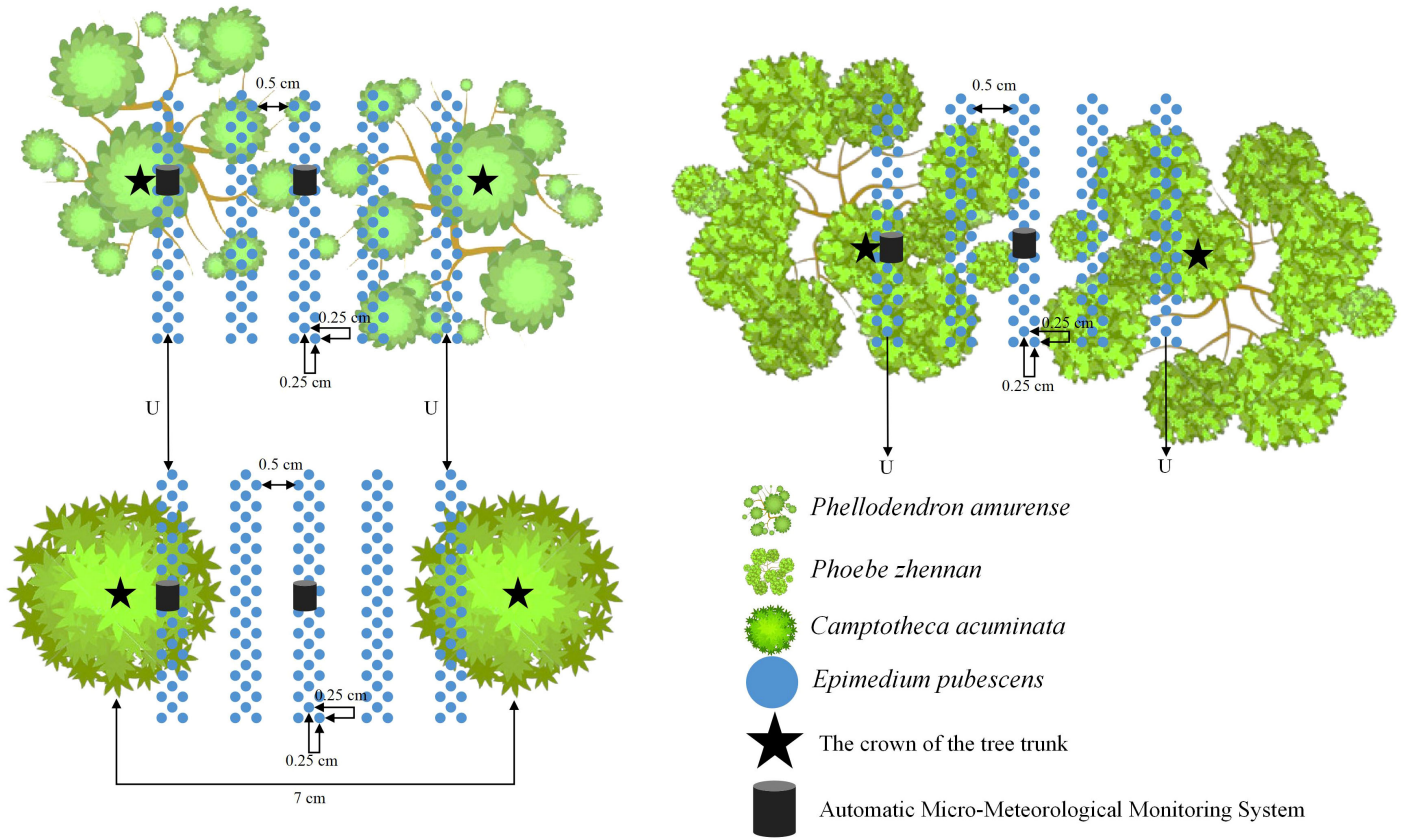
Understory cultivation of *E. pubescens* in three forest types.

In this study, a randomized complete block design was adopted. Three replicate plots of approximately equal area were established per forest stand, resulting in a total of 9 plots (3 stands × 3 replicates). Within each plot, plants were arranged at two representative planting positions: inter-row (I) and under-canopy (U). All plants were grown using a standardized ridge culture system (120 cm ridge width, 30 cm furrow width, 20 cm height) with a uniform spacing of 25 × 25 cm. Canopy density was assessed using a portable illuminance meter (Delixi, Model DLX-DTDHZDJ0504, Zhejiang, China) at 15 regularly spaced points within each replicate block for each forest type. Measurements were taken once per month from April to October under clear sky conditions, and the average value across the seven months was used as the canopy density for each forest stand. The three stands (3–4 years old) had average growing-season (April–October) understory canopy densities of 0.54 (HB), 0.82 (NM), and 0.67 (XS). Canopy distribution was uniform in the HB and XS stands, in contrast to the denser and more heterogeneous canopy of the NM stand (**Figure 1**). No significant differences in soil physicochemical properties were detected among the stands. All subsequent cultivation and field management practices were kept consistent across treatments.

### 2.3 Measurements

#### 2.3.1 Environmental variables and photosynthetic characteristics

In each of the three plantation forests, we established two microenvironment monitoring stations: one located 30 cm from the trunk and another at the midpoint between two tree rows. This resulted in a total of six monitoring points across the three forest types. At each point, an automatic microclimate monitoring system (Model SEW-2W automatic weather station, Beijing Shiyutong Technology Co., Ltd.) was installed. The system continuously recorded key environmental parameters at 10-minute intervals, including air temperature (°C), relative humidity (%), total solar radiation (W·m^-^²), and wind speed (m·s^-^¹). All data were logged and stored by an integrated i-Logger data acquisition unit. The resulting high temporal-resolution datasets were subsequently used to characterize diurnal and seasonal variations in these environmental factors across the different forest types and planting positions (under-canopy vs. inter-row).

Photosynthetic measurements were conducted once per month. To ensure comparability across forest stands and planting positions, all measurements were taken on the same clear day for the three stands. On each measurement day, diurnal courses of leaf photosynthetic traits were recorded from 08:00 to 17:00 (10 time points) using a LI-6400 portable photosynthesis system (LI-COR Inc., Lincoln, NE, USA). The LI-6400 portable photosynthesis system was equipped with a standard transparent leaf chamber (2 × 3 cm) and operated in open-flow mode without active control of CO_2_ concentration or light intensity. Compressed CO_2_-free air was supplied at a flow rate of 500 µmol·s^-^¹, allowing the chamber CO_2_ concentration to track ambient levels (approximately 400 µmol·mol^-^¹). Leaf block temperature was set to follow ambient air temperature, and photosynthetic photon flux density was provided entirely by natural sunlight. Before each measurement, leaves were allowed to fully equilibrate under ambient light conditions to accurately capture *in situ* photosynthetic rates. Measurements were taken sequentially at two planting positions—under-canopy and inter-row—within each forest. To minimize temporal lag, measurements at both positions were completed within 30 minutes per time point. At each time point, three representative *E. pubescens* plants of uniform and vigorous growth were selected per position per forest, yielding 18 measurements per time point (3 plants × 2 positions × 3 forests). Each full diurnal campaign comprised 180 measurements.

To capture the full range of understory light variability, two contrasting planting positions representing the extremes of the light gradient were selected: under the canopy (U, low light) and in the inter-row (I, high light). Measurements were taken at both planting positions: under the canopy (U) and in the inter-row (I). This resulted in seven diurnal measurement campaigns per forest over the growing season, for a total of 21 campaigns across the three forests. During each hourly measurement interval, three representative *E. pubescens* plants of uniform and vigorous growth were selected per position per forest, yielding 18 individual measurements per time point (3 plants × 2 positions × 3 forests). Ten time points were sampled per diurnal course. Daily mean values of environmental variables were calculated to compare microenvironmental conditions between planting positions within each forest on a monthly basis. For photosynthetic performance, the daily maximum net photosynthetic rate (*P*_n_-max) recorded during each diurnal campaign was used to analyze monthly differences in photosynthetic capacity between the under-canopy and inter-row positions.

#### 2.3.2 Growth dynamics of *E. pubescens*

To assess plant growth, monthly destructive harvests were conducted from April to November 2023. Within each replicate plot, three representative *E. pubescens* plants of uniform and vigorous growth were collected from each planting position (I and U) within each forest type. This resulted in a total of 18 plants per monthly harvest (3 plots × 2 positions × 3 forests), with measurements averaged at the plot level for subsequent statistical analyses. After collection, each plant was separated into aboveground (stems and leaves) and belowground (rhizomes and fine roots) components. The plant parts were placed in separate paper bags and oven-dried at 60 °C to constant weight in a forced-air drying oven (Model GZX-9070 MBE, Shanghai Boxun Industrial Co., Ltd.). The dry mass of each fraction was then determined using a balance with a precision of 0.01 g. For the purpose of this study, aboveground biomass was defined as the dry weight (g per plant) of stems and leaves, and belowground biomass as the combined dry weight (g per plant) of rhizomes and fine roots.

#### 2.3.3 Determination of total flavonol glycosides in *E. pubescens*

To quantify the content of total flavonol glycosides, leaf samples were collected from all six experimental plots (two planting positions × three forest types) in August and October 2023. From each plot, mature leaves were sampled from 30 individual plants. Two leaves were taken per plant, and the leaves from every 10 plants were pooled to form one biological replicate (20 leaves per replicate). The pooled samples were then dried at 60 °C, ground into a fine powder, and subjected to ultra-performance liquid chromatography (UPLC) analysis.

For UPLC analysis, approximately 0.2g of the dried leaf powder was accurately weighed and placed in a 50 mL conical flask. Extraction was performed by adding 20 mL of 50% ethanol and sonicating the mixture for 30 minutes at a water temperature of 50 °C using a KQ-500DE numerical control ultrasonic cleaner (Kunshan Ultrasonic Instruments Co., Ltd., China) operating at 100% power. The extract was then cooled, and the lost weight was compensated with 50% ethanol. The solution was thoroughly mixed and filtered through a 0.22 μm membrane filter prior to UPLC injection. The chromatographic separation was performed on a Waters ACQUITY UPLC system equipped with a photodiode array (PDA) detector. Separation was achieved using a BEH C18 column (2.1mm × 100mm, 1.7 μm particle size) maintained at 35 °C, with detection at 270 nm. The mobile phase consisted of water (A) and acetonitrile (B) at a flow rate of 0.3 mL·min^-^¹. The injection volume was 2 μL. Quantification was performed using an external standard method. A mixed stock solution was prepared by precisely weighing 500 μg each of epimedin A, B, and C, and 1000 μg of icariin, dissolved in methanol. A series of standard solutions were prepared by gradient dilution of the stock solution. Standard curves were constructed by plotting the peak area (Y) against the corresponding concentration (X, mg·mL^-^¹) for each compound.

As reported by Zhang et al. (2025), preliminary analysis showed no significant differences in total flavonol glycoside content across different forest types or planting positions. As the primary focus of this study is to elucidate the photosynthetic physiological basis for observed biomass variations—driven by understory environmental factors—detailed results on flavonol glycoside content are not presented in the Results section.

### 2.4 Data analysis

Initial data processing, including the calculation of means and standard errors, was performed using Microsoft Excel 2019. Statistical analyses were conducted in SPSS 26.0 (IBM Corp., USA). For comparisons between planting positions within the same forest stand: To assess the effects of planting position (inter-row vs. under-canopy) on environmental factors and photosynthetic parameters within each forest type (*P. amurense*, *P. zhennan*, and *C. acuminata*), we performed one-way analysis of variance (ANOVA). Prior to conducting ANOVA, the assumptions of normality and homogeneity of variances were tested using the Shapiro-Wilk test and Levene’s test, respectively. Data that did not meet the assumptions of normality were log-transformed before analysis. In cases where the homogeneity of variance assumption was violated even after transformation, a Welch’s ANOVA was performed, followed by Games-Howell *post-hoc* tests. Significant differences identified by ANOVA were further examined using *post-hoc* multiple comparisons (LSD).

For integrative analyses across all data: To elucidate the integrated regulatory mechanisms underlying biomass accumulation in *E. pubescens*, two complementary multivariate statistical approaches were employed using data pooled from all forest types and planting positions:

Variance Partitioning Analysis (VPA): Using the R programming environment, VPA was performed to quantify the relative contributions of environmental factors to the observed variation in photosynthetic traits across different seasonal scales.

Structural Equation Modeling (SEM): A SEM was constructed using AMOS software (IBM SPSS Amos, USA) to integrate data on environmental factors, photosynthetic physiological parameters, and biomass. This approach allowed us to disentangle the direct and indirect pathways through which these variables interact to influence final biomass yield. Model fit was evaluated using indices including CMIN/DF, CFI, IFI, and RMSEA. It should be noted that the structural equation modeling (SEM) used in this study tests the consistency of hypothesized causal structures with the observed data, rather than providing definitive proof of causation. The path coefficients indicate statistical associations and conditional dependencies consistent with our conceptual model, but cannot be interpreted as unequivocal evidence of causal relationships given the observational nature of the field data.

Pearson correlation analysis was also performed in SPSS 26.0 to explore relationships between environmental factors and photosynthetic characteristics on a diurnal scale. Figures, including bar charts, line graphs, and scatter plots, were generated using Origin 2021 (OriginLab Corporation, USA). A schematic diagram of the experimental site was created with Microsoft PowerPoint 2019.

## 3 Results

### 3.1 Diurnal variation characteristics of environmental factors and photosynthetic traits

Microclimatic monitoring revealed pronounced heterogeneity in light availability across the three forest stands, with spatial patterns that were closely related to canopy structure (see **Supplementary Table 1**; **Supplementary Figure 1–S3**). On a diurnal scale, no significant differences were detected in air temperature, relative humidity, or vapor pressure deficit (VPD) between the two planting positions (inter-row vs. under-canopy) within the same forest type. In contrast, total solar radiation showed considerable spatial variation that varied markedly among stands.

In the *Phellodendron amurense* (HB) stand, significant differences in total solar radiation between positions were observed primarily in July (**Supplementary Figure 1**). The maximum total solar radiation recorded at the inter-row (HBI) and under-canopy (HBU) positions were 218.7 ± 5.6 W·m^-^² and 218.8 ± 12.1 W·m^-^², respectively. The largest inter-position difference occurred at 15:00 in July, reaching 183.5 W·m^-^². A more persistent and pronounced pattern was evident in the *Phoebe zhennan* (NM) stand, where total solar radiation differed significantly (P < 0.05) between positions in May, July, September, and November. Throughout these months, total solar radiation at the inter-row position (NMI) consistently exceeded that under canopy (NMU). Peak total solar radiation at NMI consistently occurred around midday (13:00 or 15:00), attaining values as high as 287.5 ± 2.5 W·m^-^² in July. In sharp contrast, peaks at NMU were significantly lower, occurred earlier in the day (09:00–13:00), and never exceeded 37.3 ± 17.1 W·m^-^² (**Table 1**). Consequently, the maximum differences between NMI and NMU—up to 279.6 W·m^-^²—were recorded during these midday periods. In the *Camptotheca acuminata* (XS) stand, total solar radiation heterogeneity was far less pronounced. A significant difference between positions was detected only at 11:00 in May (F = 45.06, P < 0.01), at which time total solar radiation at the under-canopy position (XSU) was 85.5% lower than that at the inter-row position (XSI).

**Table 1:** Daily dynamics of total solar radiation at different planting positions within the same stand in July and September.

Month	Time	HBI	HBU	NMI	NMU	XSI	XSU
Jul.	8:00	10.64 ±0.10, b	98.69 ±37.11, a	25.69 ±5.26, a	7.18 ±2.12, b		
	9:00	218.74 ±5.65, a	61.96 ±7.53, b	48.27 ±6.37, a	12.18 ±1.85, b	15.12 ±1.55	7.93 ±1.07
	10:00	32.89 ±0.14	33.00 ±12.17	63.26 ±9.31, a	15.45 ±0.98, b	7.95 ±1.67	9.70 ±2.21
	11:00	32.62 ±5.00, a	15.87 ±2.68, b	224.81 ±5.70, a	12.90 ±3.50, b	29.58± 19.19	14.58 ±7.95
	12:00	63.22 ±11.93	21.88 ±3.19	260.39 ±22.73, a	6.02 ±0.17, b	8.74 ±2.66	8.14 ±0.77
	13:00	29.75 ±8.29	15.02 ±1.80	287.47 ±2.53, a	7.86 ±0.76, b	9.19 ±2.17	9.30 ±2.50
	14:00	91.18 ±19.56	130.85 ±13.16	286.00 ±5.33, a	6.41 ±1.17, b	10.77 ±3.07	9.53 ±1.59
	15:00	35.33 ±3.64, b	218.85 ±12.15, a	164.22 ±67.35, a	14.31 ±7.55, b	10.75 ±2.77	9.01 ±1.22
	16:00	156.19 ±16.04, a	12.64 ±0.09, b	33.48 ±15.48, a	11.98 ±3.38, b	3.66 ±1.55	3.37 ±0.57
	17:00	49.60 ±0.25, a	14.97 ±3.19, b	19.39 ±2.09, a	4.37 ±0.24, b		
Sep.	8:00	19.22 ±3.27	17.57 ±3.51				
	9:00	28.03 ±3.45	37.74 ±15.30	23.59 ±2.83, a	4.30 ±0.40, b	13.66 ±0.94	11.58 ±0.66
	10:00	45.54 ±21.82	38.69 ±5.16	52.99 ±6.01, a	5.45 ±1.65, b	18.07 ±0.83	18.34 ±1.88
	11:00	42.47 ±3.59	55.71 ±10.54	73.69 ±4.33, a	4.50 ±1.06, b	25.18 ±2.42	24.13 ±1.14
	12:00	54.74 ±8.91	54.22 ±8.73	92.79 ±15.61, a	4.92 ±2.42, b	23.77 ±1.33	23.82 ±1.47
	13:00	70.10 ±28.14	62.33 ±13.91	67.79 ±21.38, a	6.95 ±1.38, b	30.60 ±1.70	27.59 ±2.53
	14:00	42.16 ±13.09	49.53 ±14.62	104.51 ±32.10, a	4.76 ±1.23, b	22.91 ±1.95	24.41 ±1.98
	15:00	31.69 ±7.75	52.06 ±22.39	126.22 ±39.98, a	5.96 ±1.74, b	22.46 ±5.52	18.44 ±4.78
	16:00	19.01 ±2.39	22.68 ±5.02	45.00 ±10.40, a	3.95 ±1.05, b	9.71 ±0.73	7.16 ±0.85
	17:00	10.94 ±1.46	11.70 ±1.84			3.04 ±0.43	3.18 ±0.68

Different letters indicate significant differences among treatments in the daily-scale observations (*P* < 0.05).

**Figure 2:**
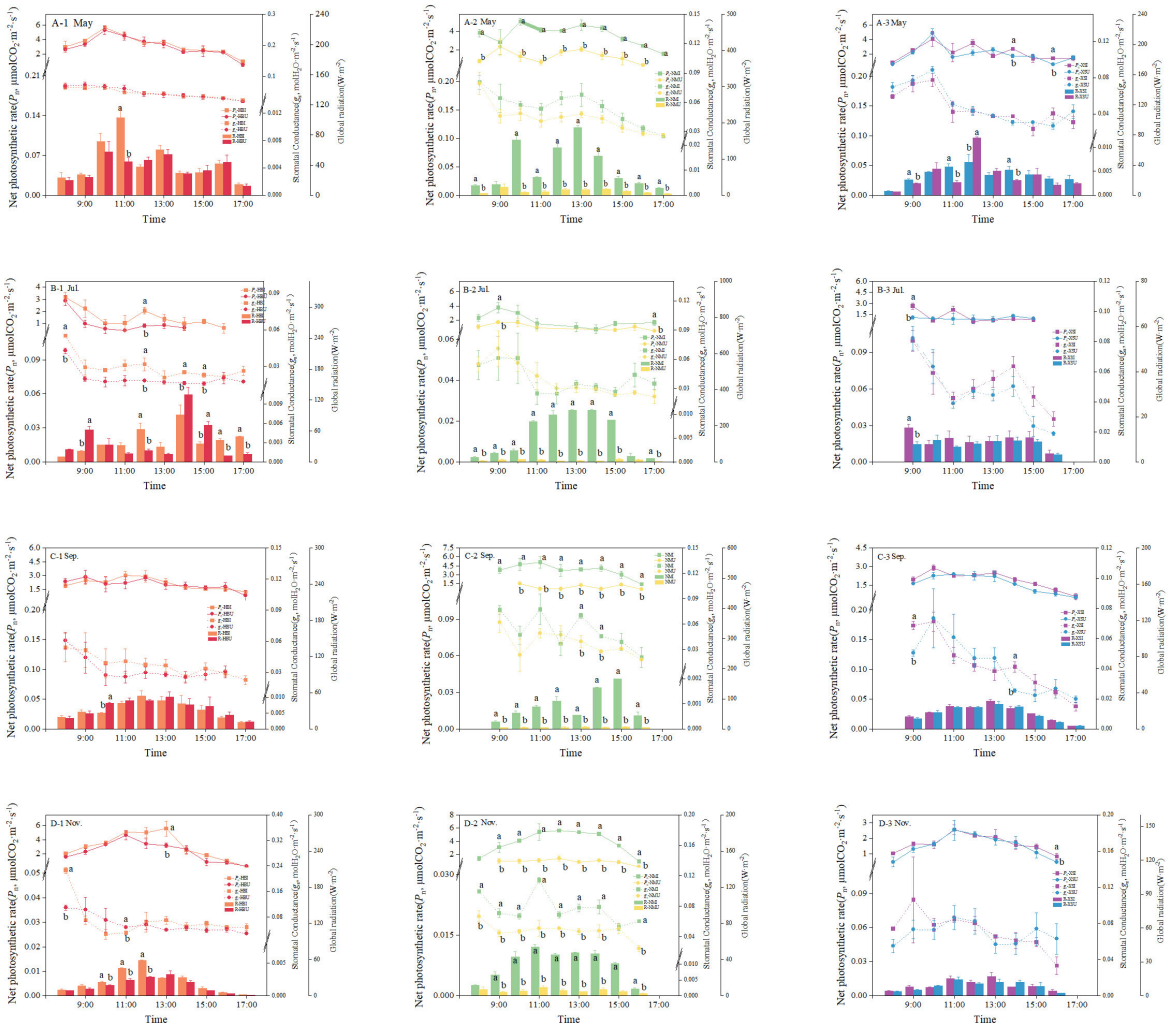
Diurnal dynamics of photosynthetic traits of *E. pubescens* at different planting positions within the same forest stand. Panels **(A–D)** represent May, July, September, and November, respectively. Subplots (A-1), (B-1), (C-1), and (D-1) correspond to the *P. amurense* stand; (A-2), (B-2), (C-2), and (D-2) to the *P. zhennan* stand; (A-3), (B-3), (C-3), and (D-3) to the *C. acuminata* stand. Different lowercase letters indicate statistically significant differences among treatments at the diurnal scale (P < 0.05).

The diurnal patterns of net photosynthetic rate (*P*_n_) and stomatal conductance (*g*_s_) in *Epimedium pubescens* under the contrasting light environments were shown in **Figure 2**. Overall, photosynthetic parameters differed only minimally—and mostly non-significantly—between planting positions within the HB and XS stands. In sharp contrast, pronounced and consistent differences were observed between the NMI and NMU positions in the dense-canopy NM stand.

Net photosynthetic rates in the HB stand showed significant positional differences only at isolated time points: at 12:00 in July (F= 16.99, P < 0.01) and 13:00 (F= 4.66, P < 0.05) in November, where *P*_n_ under the canopy (HBU) was lower than in the inter-row (HBI) by 1.25 and 2.37 μmol CO_2_·m^-^²·s^-^¹, respectively. The most striking differences emerged under the *P. zhennan* canopy. Here, *P*_n_ at the NMI position was consistently and significantly higher than at NMU across nearly all measurement times, particularly in September and November (P < 0.05; **Figure 2C-2, D-2**). In these two months, the peak differences occurred at 11:00 and 13:00, respectively. The *P*_n_ peaks in the NMI group reached 5.07 and 5.39 μmol CO_2_·m^-^²·s^-^¹, respectively, while those in the NMU group were only 0.60 and 0.88 μmol CO_2_·m^-^²·s^-^¹. In contrast, the diurnal *P*_n_ curve of the NMU group remained at a relatively low and stable level throughout the day.

A parallel trend was evident for *g*_s_, with the most pronounced and frequent significant differences again occurring in the NM stand, especially in November (**Figure 2D-2**). For the HB and XS stands, significant differences in both *P*_n_ and *g*_s_ were sporadic and confined to isolated time points (e.g., July and November for HB; May and September for XS). The absolute differences at these times were comparatively small, typically below 1.5 μmol CO_2_·m^-^²·s^-^¹ for *P*_n_ and 0.03mol H_2_O·m^-^²·s^-^¹ for *g*_s_.

### 3.2 Seasonal variation characteristics of environmental factors and photosynthetic traits

At the seasonal scale, key microclimatic variables—including air temperature, relative humidity, and vapor pressure deficit—showed no significant differences between planting positions within any given forest stand. In contrast, total solar radiation exhibited pronounced spatial heterogeneity, which varied considerably among the three forest types.

**Figure 3:**
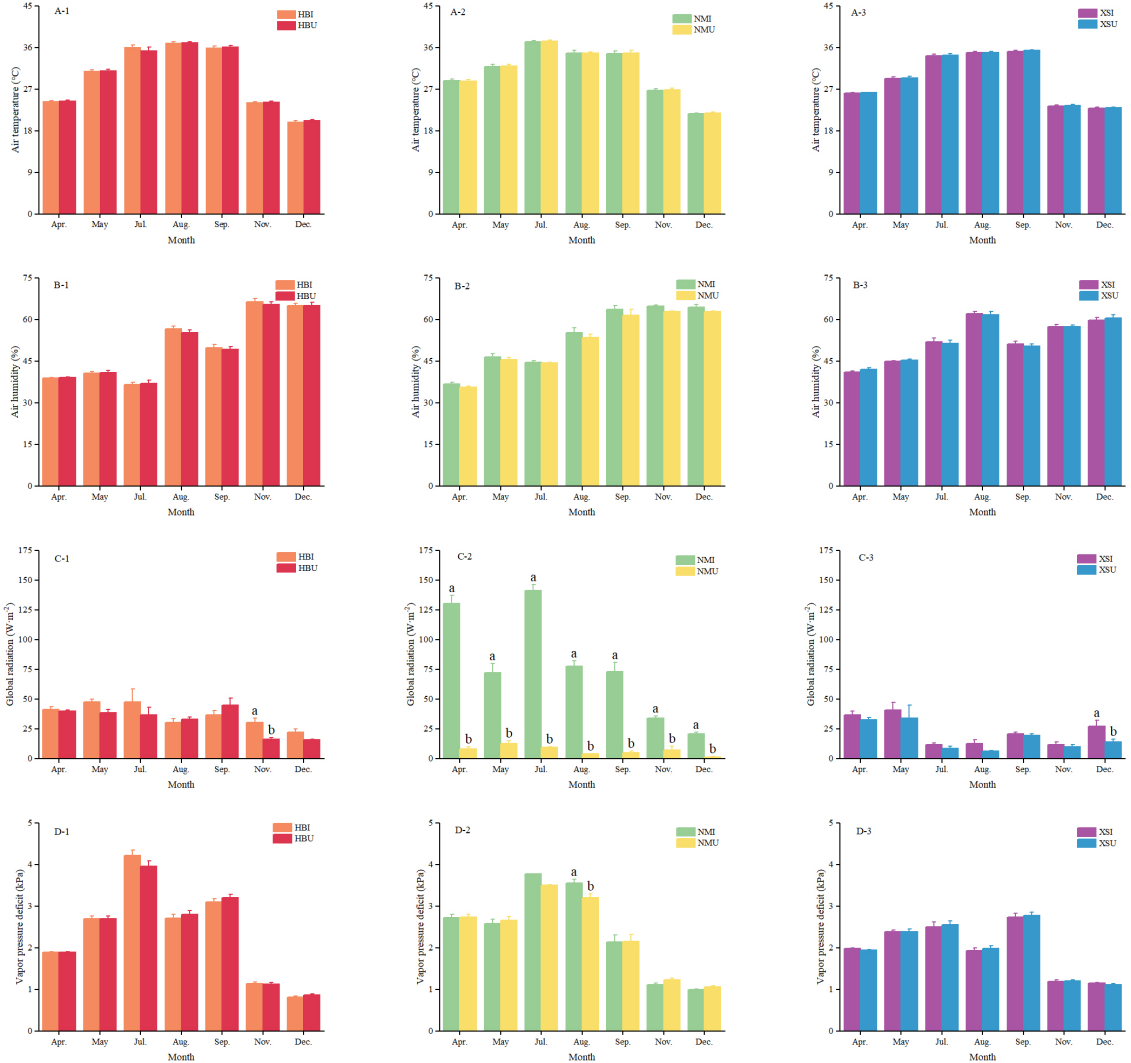
Comparison of seasonal variations in environmental factors across different planting positions within the same forest stand. Panels **(A–D)** represent air temperature, air humidity, total solar radiation, and vapor pressure deficit, respectively. Subplots (A-1), (B-1), (C-1), and (D-1) correspond to the *P. amurense* stand; (A-2), (B-2), (C-2), and (D-2) to the *P. zhennan* stand; (A-3), (B-3), (C-3), and (D-3) to the *C. acuminata* stand. Different lowercase letters indicate statistically significant differences among treatments at the seasonal scale (P < 0.05).

The most consistent and significant differences in total solar radiation were observed in the NM stand, where NMI and NMU positions differed significantly (P < 0.05) throughout the monitoring period (**Figure 3C-2**). Peak seasonal total solar radiation occurred in July at the NMI position (141.3 ± 5.1 W·m^-^²) and in March at the NMU position (20.9 ± 4.7 W·m^-^²). Consequently, the largest positional difference (131.4 W·m^-^²) was recorded in July. In the HB stand, a significant difference in total solar radiation between positions was detected only in November, during which the HBI site received 83.8% more total solar radiation than the HBU site. A similar sporadic pattern was observed in the XS stand, where the only significant positional difference occurred in December, with the XSI position receiving 94.2% higher total solar radiation than its XSU counterpart.

**Figure 4:**
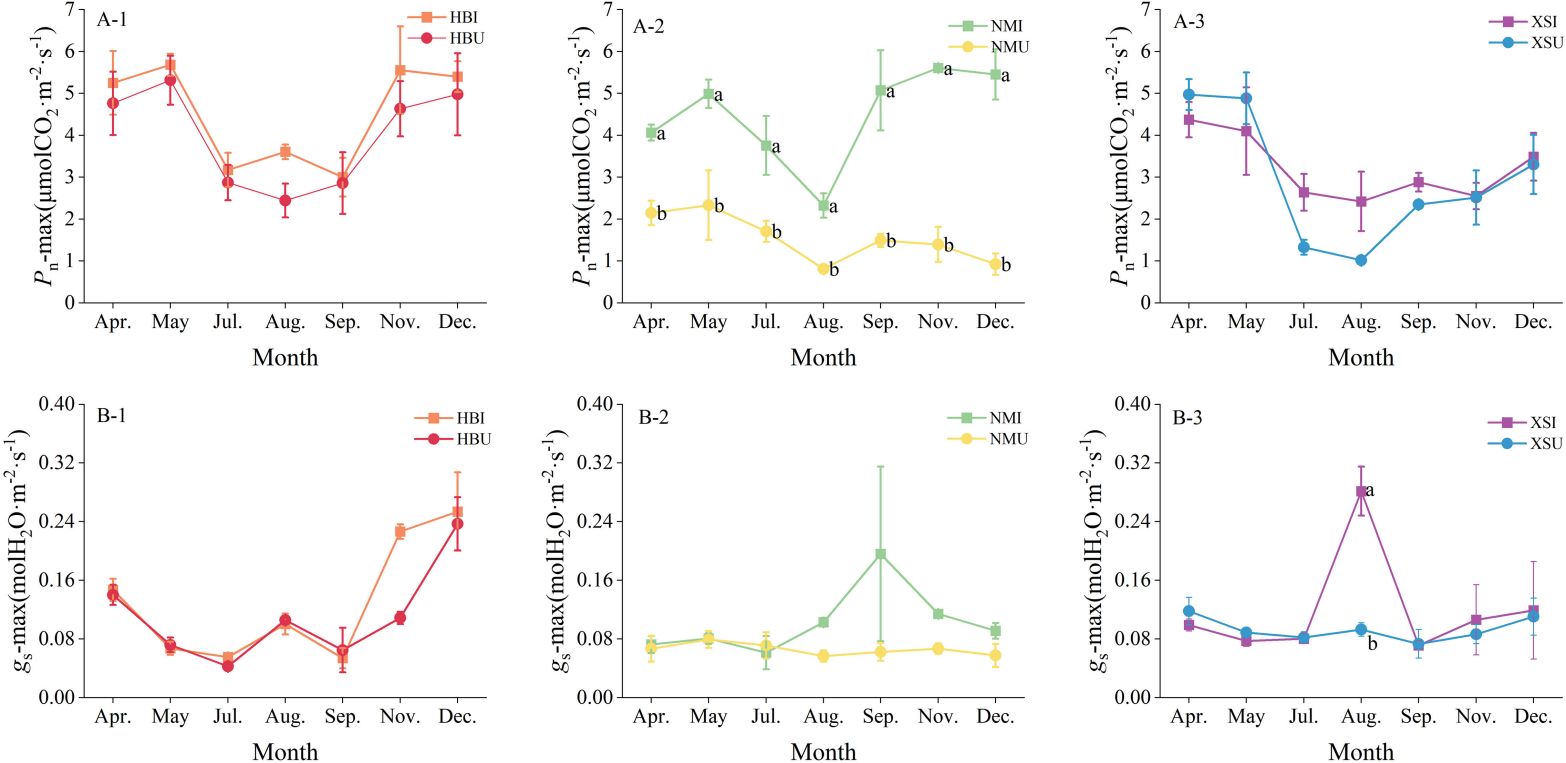
Comparison of seasonal variations in photosynthetic traits of *E. pubescens* cultivated at different positions within the same forest stand. Panels **(A, B)** represent daily maximum net photosynthetic rate and daily maximum stomatal conductance, respectively. Subplots (A-1) and (B-1) correspond to the *P. amurense* stand; (A-2) and (B-2) to the *P. zhennan* stand; (A-3) and (B-3) to the *C. acuminata* stand. Different lowercase letters indicate statistically significant differences among treatments at the seasonal scale (P < 0.05).

The seasonal patterns of daily maximum net photosynthetic rate (*P*_n_-max) and stomatal conductance (*g*_s_-max) for *E. pubescens* across the different planting positions were summarized in **Figure 4**. For *P*_n_-max, significant positional differences were confined to the NM stand, where values recorded at the I position consistently exceeded those U in all sampled months (P < 0.05). The magnitude of this difference varied seasonally, peaking in December (4.53 μmol CO_2_·m^-^²·s^-^¹) and reaching a minimum in August (1.52 μmol CO_2_·m^-^²·s^-^¹). In contrast, no significant differences in *P*_n_-max were observed between positions within either the HB or the XS stands. Significant differences in *g*_s_-max were less frequent and followed a different pattern. Notable positional differences emerged only in November for the HB stand and in August for the XS stand (F= 29.71, P < 0.05). In both instances, *g*_s_-max was significantly higher for plants located in the inter-row position compared to those under-canopy.

### 3.3 Correlation analysis between environmental factors and photosynthetic characteristics

**Figure 5:**
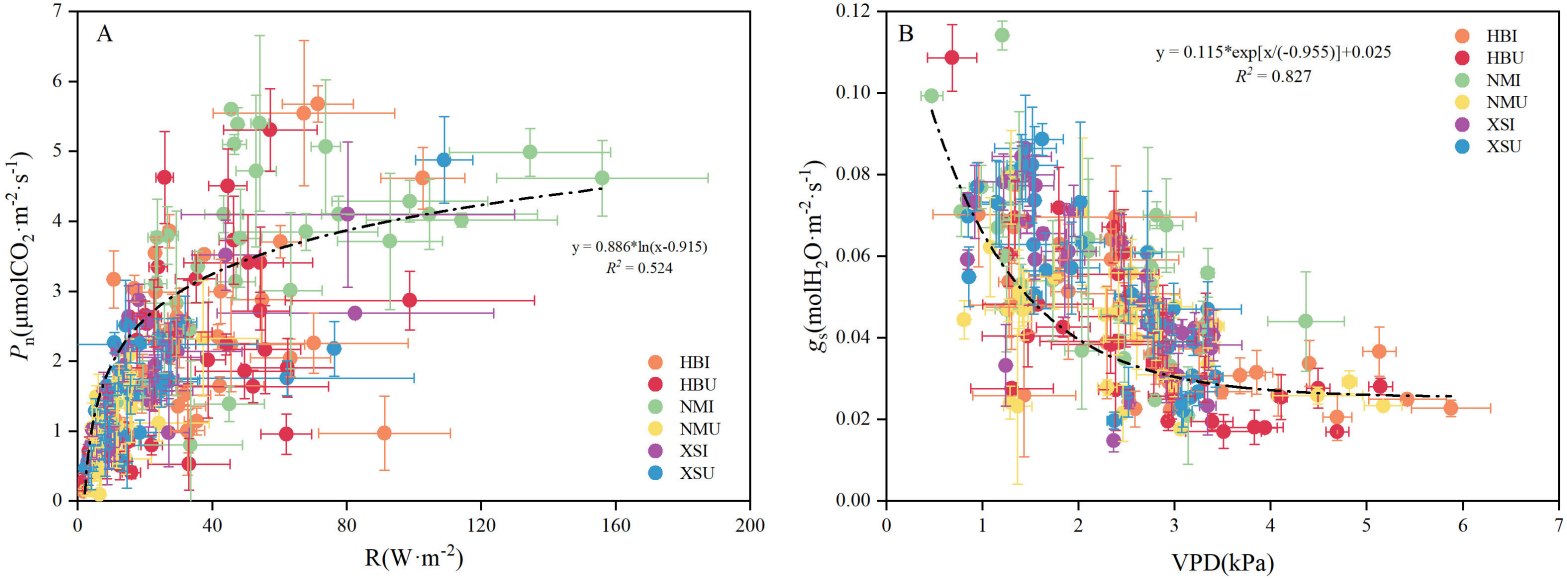
Linear regression analysis between major environmental factors and photosynthetic characteristics at the daily scale. **(A)** shows the linear regression analysis of net photosynthetic rate and total solar radiation, while **(B)** illustrates that of stomatal conductance and vapor pressure deficit. Vertical and horizontal error bars represent the standard deviation (SD) of the mean (n = 3).

Correlation analysis revealed distinct associations between the microenvironmental drivers and the key photosynthetic traits of *E. pubescens* (**Table 2**). A strong and significant positive correlation was found between *P*_n_-max and total solar radiation (r = 0.75, P < 0.05). For stomatal regulation, *g*_s_-max was significantly correlated with atmospheric moisture conditions. It showed a strong negative correlation with vapor pressure deficit (VPD) (P < 0.01) and a moderate positive correlation with relative humidity (r = 0.59, P < 0.05). The association with VPD was notably stronger (higher absolute r-value) than that with air temperature, which also showed a significant but weaker negative correlation with *g*_s_-max. Guided by the correlation results, we performed linear regression on the two variable pairs showing the strongest associations (**Figure 5**). The analysis revealed that *P*_n_ was best fitted by an exponential function of total solar radiation (R² = 0.524), whereas *g*_s_ was best fitted by a logarithmic function of vapor pressure deficit (VPD) (R² = 0.827).

**Table 2:** Correlation analysis between understory environmental factors and net photosynthetic rate and stomatal conductance at the daily scale.

PhotosyntheticParameters	Ta	RH	R	VPD
*P*_n_ (HBI)	-0.39*	0.26	0.32	-0.37*
*P*_n_ (HBU)	-0.24	0.20	0.52**	-0.27
*P*_n_ (NMI)	-0.16	0.27	0.65**	-0.24
*P*_n_ (NMU)	-0.11	-0.11	0.68**	0.03
*P*_n_ (XSI)	-0.08	0.02	0.69**	0.03
*P*_n_ (XSU)	0.00	-0.12	0.74*	0.05
*P*_n_ (Total)	-0.16*	0.07	0.75**	-0.11
*g*_s_ (HBI)	-0.70**	0.60	-0.10	-0.71**
*g*_s_ (HBU)	-0.72**	0.65	0.00	-0.76**
*g*_s_ (NMI)	-0.63**	0.56**	0.01	-0.64**
*g*_s_ (NMU)	-0.55**	0.31	0.25	-0.45*
*g*_s_ (XSI)	-0.57**	0.78**	-0.14	-0.69**
*g*_s_ (XSU)	-0.64**	0.71**	-0.16	-0.78**
*g*_s_ (Total)	-0.62**	0.59**	0.00	-0.69**

* indicates *P* < 0.05, ** indicates *P* < 0.01.

**Figure 6:**
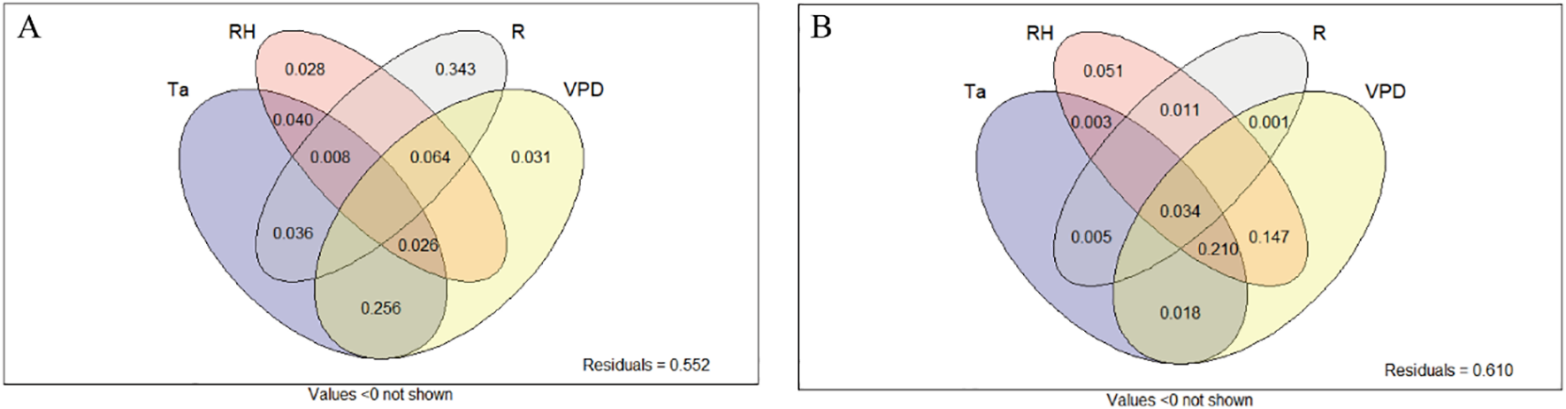
Variance partitioning analysis (VPA) of environmental factors and photosynthetic characteristics at the seasonal scale. Panels **(A, B)** represent the daily maximum net photosynthetic rate and the daily maximum stomatal conductance, respectively.

To quantify the seasonal-scale influence of microenvironmental factors on key photosynthetic traits, a variance partitioning analysis (VPA) was performed. As indicated in **Figure 6**, total solar radiation emerged as the dominant driver, explaining 34.3% of the variance in *P*_n_-max. In contrast, the independent contributions of air humidity and VPD to *P*_n_-max variance were considerably lower (2.8% and 3.1%, respectively). For *g*_s_-max, air humidity alone showed a significant independent contribution, accounting for 5.1% of the observed variance.

**Figure 7:**
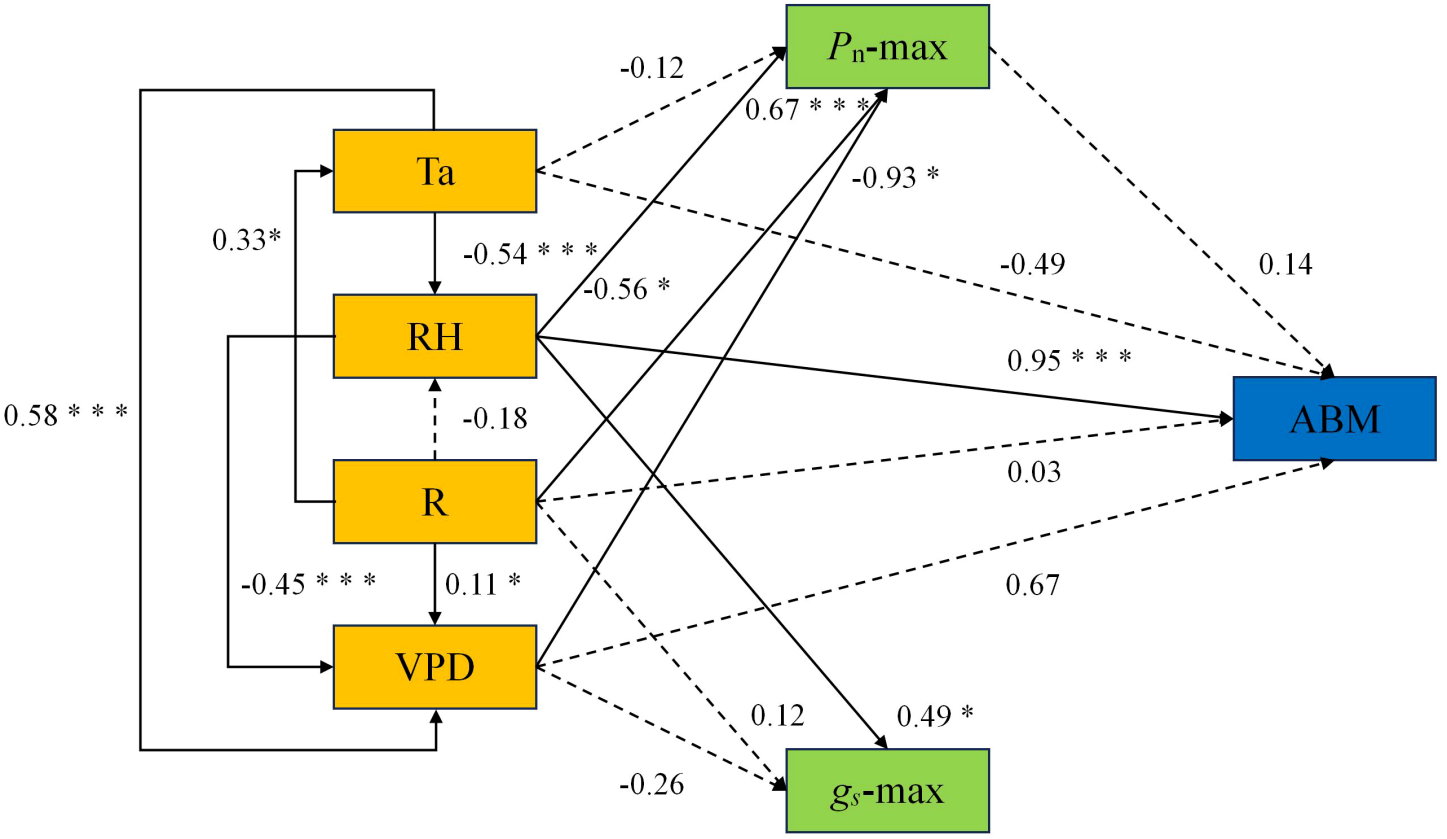
Structural equation model linking environmental factors, photosynthetic characteristics, and aboveground biomass. * indicates P < 0.05, ** indicates P < 0.01, *** indicates P < 0.001.

To elucidate the seasonal-scale interaction pathways among microenvironmental factors, photosynthetic characteristics, and aboveground biomass, we constructed a structural equation model (SEM; **Figure 7**). The model demonstrated a good fit to the observed data, with fit indices meeting recommended thresholds (CMIN/DF = 1.213, CFI= 0.996, IFI= 0.996, RMSEA= 0.080). The path analysis revealed distinct regulatory mechanisms. *P*_n_-max was co-regulated by multiple factors: it was positively influenced by solar radiation (β = 0.67, P < 0.001) but negatively affected by both relative humidity (β = -0.56, P < 0.05) and vapor pressure deficit (β = -0.93, P < 0.05). In contrast, *g*_s_-max was primarily and positively regulated by relative humidity (β = 0.49, P < 0.05). Notably, aboveground biomass (ABM) was strongly and directly driven by relative humidity, which exerted a dominant positive effect (β = 0.95, P < 0.001).

**Figure 8:**
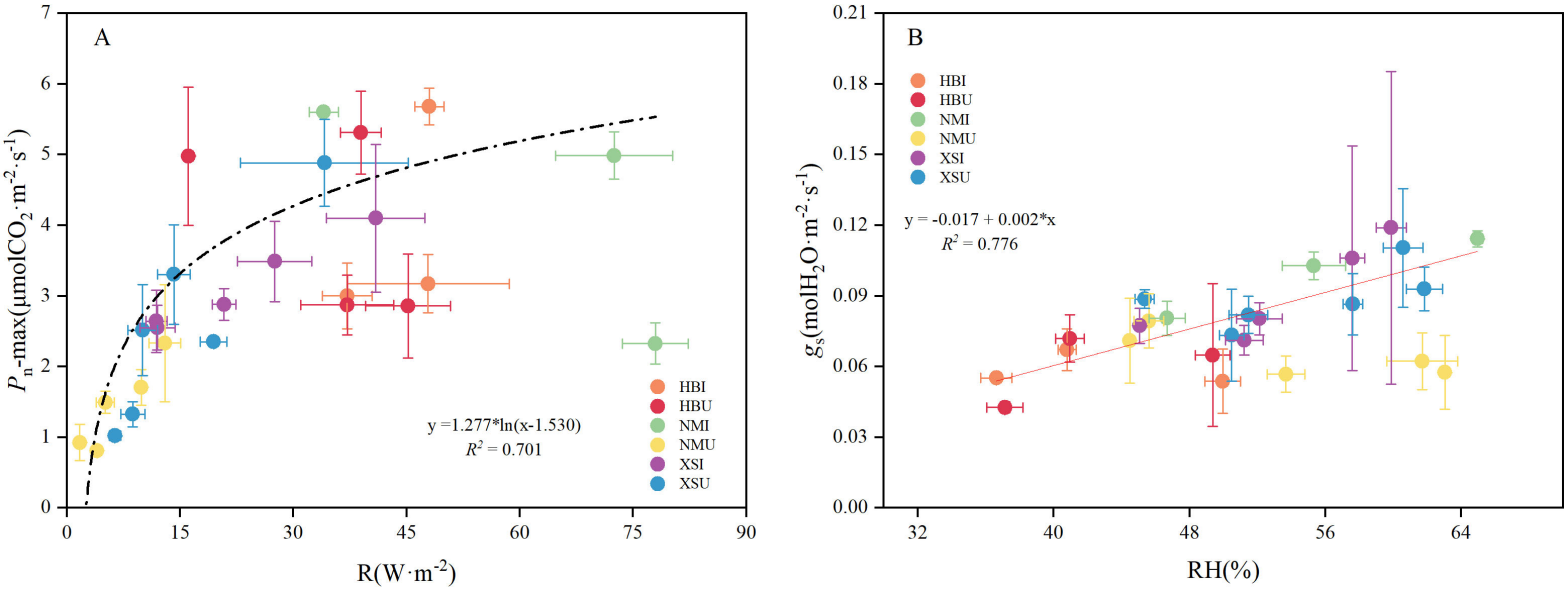
Linear regression analysis between major environmental factors and photosynthetic characteristics at the seasonal scale. **(A)** shows the linear regression analysis of daily maximum net photosynthetic rate and total solar radiation, while **(B)** illustrates the relationship between stomatal conductance and air humidity. Vertical and horizontal error bars represent the standard deviation (SD) of the mean (n = 3).

Based on the strongest seasonal correlations identified, linear regression models were fitted to the corresponding variable pairs (**Figure 8**). The results indicated that the *P*_n_-max was best modelled as a logarithmic function of solar radiation (R² = 0.701). In contrast, *g*_s_ was best described by a linear function of relative humidity (R² = 0.776).

## 4 Discussion

A key novelty of this study lies in its integration of diurnal and seasonal dynamics to capture the full complexity of photosynthetic responses of *Epimedium pubescens* to heterogeneous understory environments. In addition, by combining correlation analysis, variance partitioning, and structural equation modeling, we quantitatively disentangled the relative contributions of key environmental drivers—solar radiation, vapor pressure deficit, and air humidity—across different temporal scales. This multi-scale, multi-method approach provides new insights into how canopy structure regulates understory light heterogeneity and subsequently shapes photosynthetic performance and biomass accumulation in shade-tolerant medicinal plants.

### 4.1 Photosynthetic plasticity of *E. pubescens* to heterogeneous understory light

The understory light environment is shaped by multiple factors, including the canopy density of the overstory canopy, leaf area index, and spatial configuration of the stand (Kovács et al., 2017; Jin et al., 2024). In this study, we found no significant differences in air temperature, relative humidity, or vapor pressure deficit (VPD) between the inter-row and under-canopy positions across the three plantation forests. This suggests that within the canopy density range examined (0.54–0.82), the forest canopy appears to homogenize these microclimatic factors, a finding consistent with previous reports of low spatial variability in understory temperature and humidity in structurally intact forests (Kovács et al., 2017, 2020).

In contrast, total solar radiation exhibited pronounced spatiotemporal heterogeneity across both forest types and planting positions. The greatest and most sustained differences were observed in the *Phoebe zhennan* (NM) stand, which had the highest canopy density (0.82). Here, total solar radiation in the inter-row position consistently exceeded that under-canopy across multiple months, with a maximum difference of 279.6 W·m^-^². This suggests that canopy density story is the key structural determinant of understory light resource distribution (Jucker et al., 2018; Ma et al., 2025). The dense and unevenly distributed foliage of the NM canopy creates a pronounced mosaic of sunflecks and shade, amplifying the contrast between the inter-row position (influenced by lateral and some direct beam radiation) and the heavily shaded under-canopy position. In comparison, the more uniformly structured canopies of the *Phellodendron amurense* (HB, canopy closure 0.54) and *Camptotheca acuminata* (XS, 0.67) stands resulted in lower light heterogeneity, with significant positional differences occurring only during specific seasons. This aligns with findings from tropical secondary forests, where canopy attributes is the primary driver of understory light heterogeneity (Matsuo et al., 2021). Similarly, studies in Pinus massoniana plantations have shown that gap size significantly influences light availability, with larger gaps consistently providing higher photosynthetic photon flux density (Wang et al., 2025). Collectively, these results underscore that canopy density and uniformity are key factors governing the spatiotemporal distribution of solar radiation within forests.

Plants can acclimate to heterogeneous light environments by adjusting their photosynthetic physiology (Yan et al., 2008; Retkute et al., 2015; Zhang et al., 2024). In the NM stand, where light heterogeneity was most pronounced, the net photosynthetic rate (*P*_n_) of *E. pubescens* plants in the inter-row position was significantly higher than that of under-canopy plants during most measurement periods across all months, with a maximum difference of 4.51 μmol CO_2_·m^-^²·s^-^¹. This is consistent with a sensitive and positive photosynthetic response to increased light availability, likely involving enhanced light capture and carbon assimilation efficiency. Conversely, in the HB and XS stands—where light distribution was more uniform—photosynthetic parameters differed only minimally and sporadically between positions. This suggests that when light resources are evenly distributed, photosynthetic performance converges. Our results align with findings for *P. zhennan* seedlings, whose photosynthetic traits (e.g., specific leaf area, chlorophyll content, photosynthetic efficiency parameters) differed significantly under different light regimes created by thinning (Su et al., 2024). Similarly, maple seedlings have been shown to adjust key photosynthetic parameters, such as light-saturated photosynthetic rate, along light intensity gradients (Zhang et al., 2022).

Notably, even under the stark light gradient in the NM stand, the *P*_n_ of under-canopy plants did not drop to zero but remained at a low, relatively stable level. This reflects an adaptive strategy typical of shade-tolerant medicinal plants: under prolonged shade, plants can maintain basal metabolism through physiological adjustments such as increasing photosynthetic pigment content, optimizing photosystem II efficiency, and lowering the light compensation point (Liu et al., 2016; Yang et al., 2020; Lu et al., 2021). However, this adaptation comes at the cost of reduced growth, as evidenced by the significantly lower biomass accumulation of under-canopy plants compared to their inter-row counterparts. Stomatal conductance (*g*_s_) generally followed a similar response pattern to *P*_n_, but showed more pronounced positional differences at certain times (e.g., November in the NM stand), suggesting that stomatal regulation may play a role in the plant’s response to combined variations in light and water availability.

Our field results corroborate earlier pot experiments, which indicated that moderate light levels are optimal for photosynthesis and growth in *E. pubescens* (Pan et al., 2021; Ma et al., 2023; Zhang et al., 2025a). However, the present study further reveals that in real, dynamic forest understories, absolute light intensity is not the sole determinant. Rather, the spatiotemporal uniformity of light distribution, coupled with its interaction with other microenvironmental factors (e.g., humidity, VPD), jointly shapes the ultimate physiological performance and productivity of the plants. This integrated perspective echoes findings in wheat-tree agroforestry systems, where the impact of light reduction on crop yield must be evaluated in conjunction with concurrent changes in other microclimatic variables (Jia et al., 2021).

### 4.2 Interactive effects of stand type and planting position on productivity

The productivity of agroforestry systems is governed not only by the physiological traits of the understory crop but also by the dynamic interactions between the overstory trees and the understory plants (Dupraz et al., 2019; Yang et al., 2021; Bhardwaj et al., 2024; Tosh et al., 2024). This study demonstrates that optimal planting configurations are not universally applicable but are instead highly dependent on the specific structural characteristics of the forest canopy.

For stands with dense canopies, low light transmittance, and high spatial heterogeneity in light—such as the *P. zhennan* (NM) plantation—the inter-row planting strategy effectively avoids severe shading directly under-canopy. It allows plants to utilize the relatively abundant lateral and transmitted light available in the inter-row spaces, thereby significantly enhancing the photosynthetic efficiency and potential yield of *E. pubescens*. This finding provides a clear practical guideline for managing medicinal plants in similarly high-closure forests: actively optimizing spatial configuration by placing plants in microhabitats with superior light conditions is a key strategy for alleviating light limitation and improving system productivity. Parallel evidence exists for other species; for example, in jujube-cotton intercropping systems, an appropriate tree spacing (e.g., 0.5m) can improve the light environment, increase the net photosynthetic rate of cotton, and ultimately boost yield by 18.2% compared to monoculture (Wang et al., 2022).

In contrast, in stands with relatively open and uniformly structured canopies, such as *P. amurense* (HB) and *C. acuminata* (XS), planting position had a minor influence on the photosynthetic physiology of *E. pubescens*. Notably, our data show that *E. pubescens* maintained relatively high photosynthetic efficiency even under the XS canopy (closure 0.67). This suggests the existence of an optimal canopy closure window. Within this range, the canopy provides sufficient shading to mitigate potential photoinhibition from intense summer sunlight while simultaneously ensuring adequate and uniformly distributed understory light. Such conditions are associated with higher photosynthetic production and biomass accumulation in shade-tolerant plants like *E. pubescens*. This observation aligns with findings in an agroforestry system integrating Gleditsia sinensis and Polygonatum cyrtonema, where the latter achieved optimal biomass accumulation under a light transmittance range of 32–72% (Gao et al., 2021).

It should be noted, however, that even in stands with uniform canopies where photosynthetic parameters showed no significant positional differences, spatial heterogeneity in biomass may still occur, as observed in certain months under the HB and XS stands in this study. As belowground conditions such as soil moisture, nutrient availability, and root competition were not directly measured in this study, this heterogeneity may be associated with belowground resource dynamics or spatial variation in root distribution (Bhardwaj et al., 2024). The importance of dynamic tree-crop interactions in belowground resource acquisition is similarly emphasized by process-based models such as Hi-sAFe (Dupraz et al., 2019). Given this limitation, future research could benefit from integrating belowground processes to further explore the mechanisms underlying resource competition and complementarity in forest-herb intercropping systems.

In summary, this study suggests that differentiated planting and management strategies should be adopted based on canopy structure. For stands with dense, heterogeneous canopies (e.g., NM), optimizing spatial configuration through inter-row planting is critical. For stands with uniform canopies, management should focus on maintaining canopy closure within the suggested optimal range under the studied conditions (approximately 0.6–0.7) through practices such as selective pruning and density regulation. This targeted approach is essential for maximizing both the ecological and economic benefits of forest-herb intercropping systems.

### 4.3 Limitations and future directions

Several methodological considerations should be acknowledged when interpreting the findings of this study. First, this study focused primarily on aboveground environmental drivers (solar radiation, air temperature, relative humidity, VPD) and their effects on photosynthetic characteristics and biomass accumulation. Belowground factors—including soil moisture dynamics, nutrient availability, and root competition between *E. pubescens* and the overstory trees—were not directly measured. This omission may partially limit the interpretation of biomass variation observed in this study. For instance, even in stands with uniform light distribution (e.g., HB and XS), spatial heterogeneity in biomass was occasionally observed despite similar photosynthetic rates between planting positions, which could be attributable to belowground resource competition not captured by our aboveground-focused measurements.

Second, the observational nature of this field study precludes definitive causal inference. While structural equation modeling (SEM) was employed to explore hypothesized relationships among variables, the path coefficients indicate statistical associations consistent with our conceptual model but cannot be interpreted as unequivocal evidence of causation. Third, the findings are specific to the three forest types, climatic conditions (subtropical monsoon climate), and plant material (GT-1 clonal line) used in this study. Generalization to other Epimedium species, different canopy structures, or contrasting ecological contexts should therefore be made with caution.

Despite these limitations, this study provides a robust physiological and ecological basis for optimizing canopy light management and precision cultivation of *E. pubescens* in artificial forests. Future research should build on these findings by: (1) incorporating direct measurements of canopy structural traits (e.g., leaf area index, leaf angle distribution) to better characterize light heterogeneity; (2) integrating belowground resource monitoring (soil moisture, nutrient availability, root distribution) to comprehensively assess resource competition and complementarity; and (3) testing the applicability of these findings across a broader range of forest types, canopy densities, and geographical regions to validate their generalizability.

## Data Availability

The original contributions presented in the study are included in the article/**Supplementary Material**. Further inquiries can be directed to the corresponding author.
