# Supplementary material for: Spatiotemporal response of photosynthetic characteristics in Epimedium pubescens to understory environmental factors across three agroforestry systems
Source: Front Plant Sci. 2026 May 5;17:1796904. doi: 10.3389/fpls.2026.1796904 (PMC13184557; doi:10.3389/fpls.2026.1796904)
Supplement: Supplementary file 1 [file SupplementaryFile1.docx]

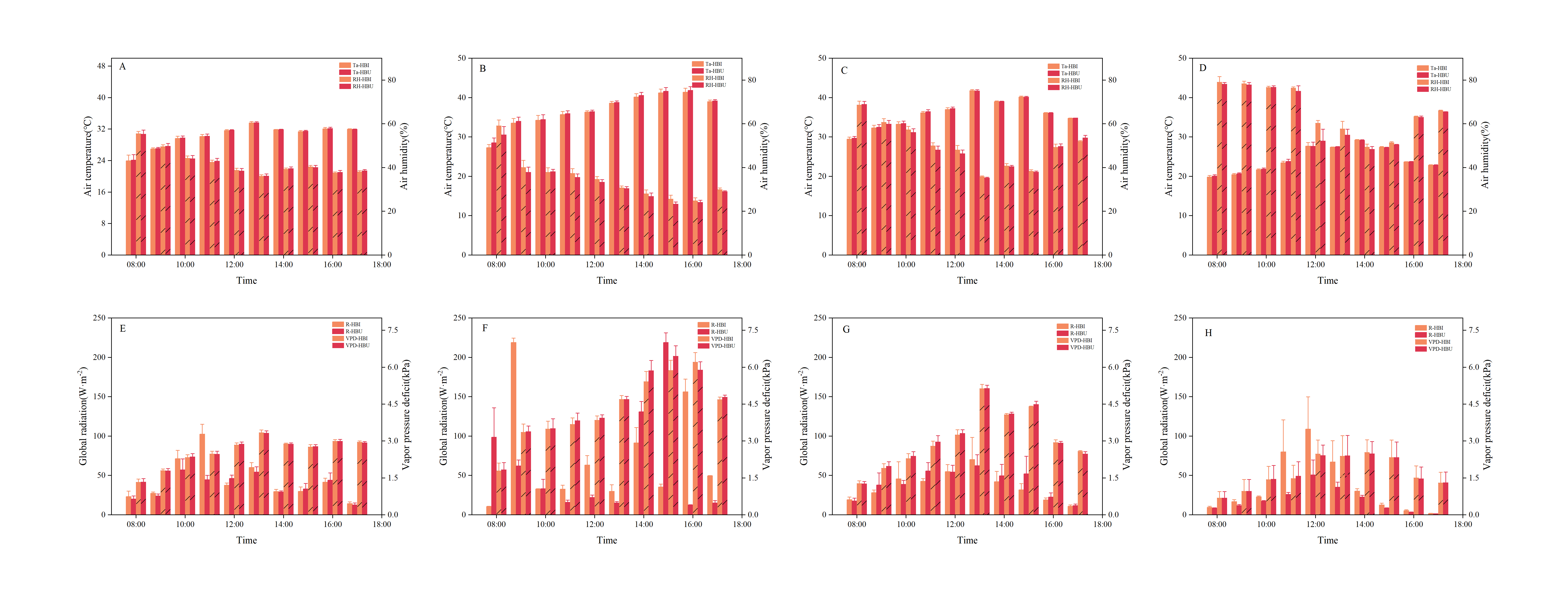
Figure S1. Comparison of daily dynamics of environmental factors at different planting positions under the *Phellodendron amurense* stand. A, B, C, and D represent May, July, September, and November, respectively; while E, F, G, and H correspond to May, July, September, and November, respectively.


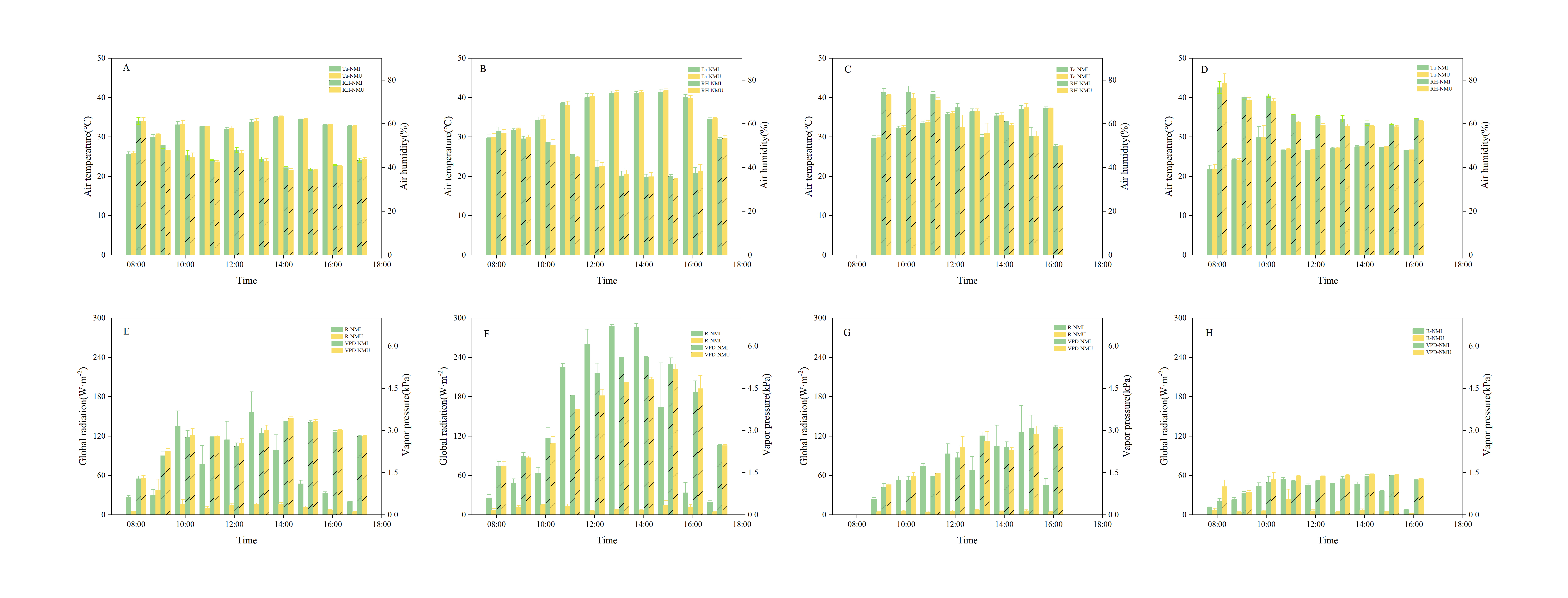


Figure S2. Comparison of daily dynamics of environmental factors at different planting positions under the *Phoebe zhennan* stand. A, B, C, and D represent May, July, September, and November, respectively; while E, F, G, and H correspond to May, July, September, and November, respectively.


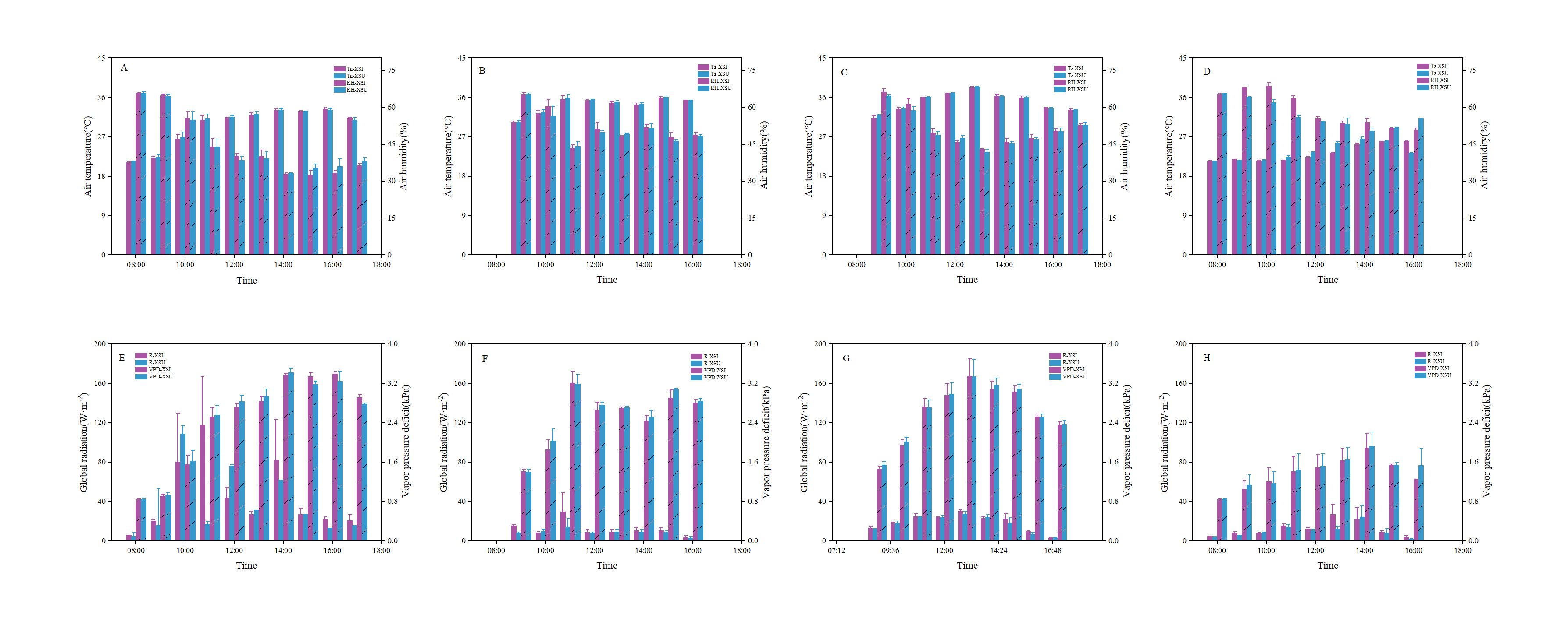


Figure S3. Comparison of daily dynamics of environmental factors at different planting positions under the *Camptotheca acuminata* stand. A, B, C, and D represent May, July, September, and November, respectively; while E, F, G, and H correspond to May, July, September, and November, respectively.

Table S1. Comparison of daily dynamics of total solar radiation at different planting positions within the same stand in May and November.

| Month | Time | HBI | HBU | NMI | NMU | XSI | XSU |
| --- | --- | --- | --- | --- | --- | --- | --- |
| May | 8:00 | 23.11±7.06 | 20.08±3.88 | 26.62±3.25 | 5.23±0.48 | 5.53±0.41 | 4.69±3.36 |
|  | 9:00 | 27.22±1.87 | 23.84±2.73 | 29.29±9.57 | 37.26±17.09 | 20.67±1.10 | 15.70±37.86 |
|  | 10:00 | 71.38±10.58 | 57.19±13.92 | 134.45±23.94 | 15.87±7.23 | 80.27±49.58 | 108.94±8.48 |
|  | 11:00 | 102.59±12.52 | 44.58±5.64 | 77.55±28.32 | 10.36±2.81 | 118.33±48.40 | 17.15±2.70 |
|  | 12:00 | 37.60±2.89 | 46.27±4.25 | 114.12±28.40 | 14.94±2.82 | 43.94±9.98 | 76.27±1.23 |
|  | 13:00 | 60.24±6.07 | 54.2±6.86 | 155.95±31.48 | 15.31±2.85 | 26.87±3.15 | 31.89±3.36 |
|  | 14:00 | 29.46±2.73 | 28.87±1.56 | 98.70±23.11 | 16.16±2.41 | 82.49±41.07 | 62.17±37.86 |
|  | 15:00 | 29.74±5.63 | 32.72±7.14 | 46.98±5.84 | 11.48±2.03 | 27.03±6.01 | 27.10±8.48 |
|  | 16:00 | 41.55±4.87 | 44.05±9.10 | 33.19±1.80 | 6.94±1.02 | 21.75±2.64 | 13.40±2.70 |
|  | 17:00 | 14.07±2.45 | 12.49±2.51 | 19.92±1.08 | 4.20±0.68 | 21.24±5.19 | 15.66±1.23 |
| Nov. | 8:00 | 9.55±1.40 | 8.4±0.41 | 11.49±0.43 | 7.16±2.73 | 4.36±0.20 | 3.90±0.31 |
|  | 9:00 | 16.78±2.31 | 11.68±1.51 | 23.16±3.31 | 4.23±0.40 | 7.90±1.52 | 5.24±0.58 |
|  | 10:00 | 22.90±1.09 | 17.25±0.94 | 43.33±5.58 | 5.39±1.59 | 7.60±0.42 | 8.59±0.63 |
|  | 11:00 | 79.77±40.88 | 25.80±2.63 | 54.10±2.60 | 24.05±14.96 | 15.35±2.28 | 14.17±2.49 |
|  | 12:00 | 108.89±40.94 | 50.61±19.12 | 45.57±1.58 | 6.10±1.50 | 11.96±2.03 | 10.87±0.91 |
|  | 13:00 | 67.21±27.01 | 35.16±6.20 | 47.58±0.43 | 4.49±0.49 | 27.06±9.46 | 12.13±2.67 |
|  | 14:00 | 30.20±2.91 | 22.58±2.30 | 46.50±3.54 | 6.79±1.13 | 22.04±12.13 | 24.77±11.43 |
|  | 15:00 | 12.64±2.04 | 8.68±0.30 | 35.67±0.92 | 4.58±1.00 | 8.52±1.74 | 8.26±2.81 |
|  | 16:00 | 5.34±0.93 | 3.33±0.37 | 7.82±0.87 | 2.47±0.28 | 4.21±1.19 | 2.26±0.22 |
|  | 17:00 | 1.69±0.26 | 1.35±0.09 |  |  |  |  |

Table S2. Comparison of aboveground biomass of *E. pubescens* at different locations within the same forest stand during the main growing season.

| Month | HBI | HBU | NMI | NMU | XSI | XSU |
| --- | --- | --- | --- | --- | --- | --- |
| May | 1.30±0.28 | 1.52±0.29 | 5.25±1.49 | 7.53±1.19 | 4.69±0.22 | 4.96±0.25 |
| Jul. | 16.90±0.21 | 2.92±0.46 | 14.23±1.49 | 10.08±2.29 | 14.16±2.58 | 14.19±1.97 |
| Sep. | 16.86±1.65 | 14.19±1.67 | 15.83±1.51 | 6.91±0.85 | 13.82±1.84 | 10.79±1.10 |
| Nov. | 29.94±1.90 | 15.72±2.88 | 32.68±2.61 | 16.60±3.83 | 26.70±5.32 | 13.75±2.6 |
| Dec. | 29.69±0.32 | 15.61±2.96 | 33.76±2.26 | 16.31±3.42 | 27.41±5.20 | 13.61±2.33 |
